# Open, Hybrid and Endovascular Management of Aortic Arch Aneurysms: Recent Updates and Future Directions

**DOI:** 10.3390/jcm15114272

**Published:** 2026-06-01

**Authors:** Dhruv R. Patel, Mohamed N. Elshabrawi, Mohammed Rahouma, Akshay Kumar

**Affiliations:** 1 NYU Grossman School of Medicine, New York, NY 10016, USA; 2 Faculty of Medicine, Port Said University, Port Said 42526, Egypt; mohamedshabrawi86@gmail.com; 3 Department of Cardiothoracic Surgery, Weill Cornell Medicine, New York, NY 10065, USA; 4 Department of Cardiothoracic Surgery, NYU Langone Health, New York, NY 10016, USA

**Keywords:** aortic arch aneurysm, endovascular treatment, hybrid technique

## Abstract

Once considered a surgical frontier fraught with risk, aortic arch aneurysms now represent a domain of evolving innovation. Despite their rarity, they pose severe risks of dissection, rupture, and mortality if not adequately managed. Primarily caused by prior aortic dissections, atherosclerosis, or connective tissue disorders, these aneurysms are often found incidentally on CTA or MRA imaging. Medical management focuses on reducing aortic wall stress through blood pressure control, risk factor modification, and regular imaging to monitor growth. Surgical intervention is typically indicated when the aneurysm diameter exceeds 5.5 cm, exhibits rapid growth, or causes symptoms such as compression or dissection. Open repair remains the gold standard for treatment due to its superior long-term outcomes, though hybrid and endovascular approaches are favored for high-risk patients due to reduced perioperative morbidity. Innovations in hybrid techniques and endovascular devices, alongside advancements in cerebral perfusion strategies, are shaping the future of personalized and minimally invasive approaches to aortic arch repair. This comprehensive review delves into the current management strategies for these aneurysms.

## 1. Introduction

Isolated aortic arch aneurysms (i.e., aortic arch with a diameter ≥ 4.5 cm) are relatively rare, with most arch aneurysms occurring as an extension of adjacent aortic aneurysms [1]. Without adequate management, aneurysms may enlarge over years, progressively increasing the risk of aortic dissection and rupture. Surgical intervention to repair these aneurysms can mitigate much of this risk, but carries its own risks and potential complications. The current review focuses on epidemiology, etiopathogenesis, and various treatment strategies for isolated arch aneurysms with their role in the current era. Of note, this article is a narrative review of aortic arch aneurysms. This approach was chosen to allow for a comprehensive synthesis of a highly heterogeneous body of literature, including small retrospective studies, surgical series, hybrid approaches, and evolving operative techniques. The wide variability in patient populations, interventions, and outcome reporting limits the feasibility and validity of a systematic review or meta-analysis.

Aneurysms of the thoracic aorta are estimated to have an incidence of 10 cases per 100,000 person-years; 10% of these aneurysms involve the aortic arch, most commonly as an extension of ascending or descending aortic aneurysms [2,3]. Isolated aortic arch aneurysms themselves are rare, with one study identifying only six isolated arch aneurysms in 780 patients with thoracic aortic aneurysms [4]. Prevalence increases with age and peaks in the 60–70 age group, with men affected more commonly than women [3]. The most common cause of arch aneurysms is a previous aortic dissection, with only an estimated 30% of arch aneurysms arising de novo [5]. Other common causes of these aneurysms are summarized in Table 1 [6,7].

Left untreated, aortic aneurysms can lead to aortic dissection, rupture, and mortality; studies have found the critical point of rupture for arch aneurysms to be a diameter of 6 cm [8,9]. The growth of aortic arch aneurysms is a slow process, with reported mean growth rates ranging from 0.07 to 0.25 cm per year [10]. Importantly, the initial aortic diameter is a risk factor for growth, with larger aneurysms growing progressively faster than smaller aneurysms [11]. In all, aortic arch aneurysm size and growth >0.55 cm/year have been found to be significant predictors of aortic rupture and mortality [5,12]. This makes the timely identification and appropriate management of aortic arch aneurysms all the more crucial.

## 2. Diagnostic Workup

Aortic arch aneurysms usually do not cause symptoms and are often discovered incidentally upon imaging. If sufficiently large, however, they can exert a local mass effect and produce symptoms including cough, wheezing, and shortness of breath due to tracheal compression, dysphasia due to esophageal compression, or hoarseness and vocal pitch changes due to recurrent laryngeal nerve compression [5]. Imaging modalities include chest X-rays, echocardiography, computed tomography angiography (CTA), and magnetic resonance angiography (MRA).

Though chest X-rays can show evidence of large aortic arch aneurysms with signs including widening of the mediastinal silhouette and aortic knob enlargement, they may be normal in the setting of small (or even large) aneurysms [2]. Hence, they are not routinely used for the evaluation of the aorta and its aneurysms. Transthoracic echocardiography (TTE) is effective at evaluating the aortic root and may be used for initial evaluation at the bedside, but may not visualize the distal ascending aorta and proximal arch well due to left mainstem bronchus interposition [13]. TTE is, however, often used for the evaluation of aortic arch pathologies in pediatric patients [14]. While transesophageal echocardiography (TEE) can visualize the entire aorta well, it is less preferred due to its invasive nature [2].

CTA and MRA are the preferred imaging modalities for the evaluation of aortic arch aneurysms. MRA is preferred if the aneurysm is suspected to involve the aortic root, as it visualizes the root better than CTA [2]. Both of these modalities, however, should be used with caution in patients with impaired kidney function as intravenous contrast dye is used in CTA and gadolinium in MRA [3]. Table 2 summarizes the utility of the various imaging modalities in the diagnosis of aortic arch aneurysms [15,16,17,18,19].

If an aneurysm is detected, further imaging is recommended to evaluate the entire aorta and major branches for concomitant aneurysms and pathologies [20]. Coronary angiography is recommended in patients who require open surgery to evaluate whether a simultaneous cardiac procedure is needed [3,21]. Likewise, molecular genetic testing is generally recommended for individuals who are younger than 60 years old, have syndromic features (e.g., Marfan or Loeys-Dietz), or have a family history of aortic disease, sudden death, or aneurysms [21,22]. Moreover, first-degree relatives of such patients are also advised to undergo genetic testing to screen for pathogenic mutations [21].

## 3. Surveillance, Medical Management, and Interventions

The goal of managing and treating aortic arch aneurysms is, intuitively, to minimize the risk of adverse events including aortic dissection and rupture. Hence, medical therapy and surveillance remain the initial steps in management of aortic arch aneurysm, with risk stratification of patients being of utmost importance. Studies have found ‘hinge points’ in aortic diameter, which are threshold diameters beyond which the rates of dissection or rupture dramatically increase; these hinge points lie at both 5.5 cm and 6 cm for the ascending aorta and at 7 cm for the descending aorta [4,20]. While no established hinge point exists for the aortic arch given the relative rarity of isolated aortic arch aneurysms, surgical intervention is generally indicated when the aneurysm is ≥5.5 cm [23].

### 3.1. Medical Management and Surveillance

The primary goal of medical management is to reduce stress on the aneurysmal wall of the aorta, primarily by reducing blood pressure and contractility [24]. Controlling atherosclerotic risk factors may also slow aneurysm growth and lower the likelihood of dissection or rupture [3]. To that end, the 2024 ESC guidelines recommended blood pressure control with a goal of less than 130/80 140/90 mmHg using beta adrenergic-blockers, angiotensin-converting enzyme inhibitors, or angiotensin receptor blockers [13]. Beta adrenergic blockade, in particular, serves as the foundation of medical therapy for patients with Marfan syndrome [25]. Guidelines also recommend smoking cessation and controlling LDL cholesterol to below 55 mg/dL with a statin [13]. For isolated aortic arch aneurysms with a diameter less than 4.0 cm, the 2010 AHA guidelines for thoracic aortic disease recommended surveillance imaging at 12-month intervals using either CT or MRI to evaluate for aneurysm growth [25]. For aneurysms with diameters exceeding 4.0 cm, the guidelines recommended surveillance imaging at 6-month intervals with either CT or MRI [25]. Table 3 summarizes the medical management recommendations.

### 3.2. Guidelines on Surgical Management

**Figure 1:**
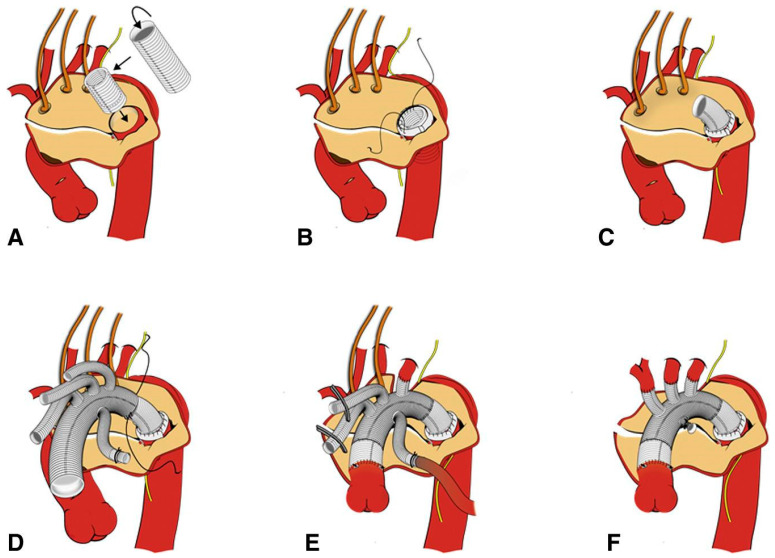
(**A**–**F**) Operative steps of an approach to performing total aortic arch replacement. Reprinted with permission from Minatoya et al. [27]. 2019, Elsevier.

Multiple considerations influence the decision to surgically intervene on an aortic arch aneurysm, including aneurysm diameter, rate of growth, and the presence of symptoms. Guidelines also emphasize shared decision-making and highlight the importance of institutional volume and aortic team expertise in patient outcomes [2]. The 2024 EACTS/STS, 2022 ACC/AHA, and 2024 ESC guidelines largely provided overlapping and complementary recommendations for the surgical management of aortic arch aneurysms. Broadly, surgical treatment is recommended for patients who are symptomatic from the aneurysm (EACTS/STS guidelines: Class 1 recommendation, Level C evidence) or with an aneurysm diameter of ≥5.5 cm (EACTS/STS: Class IIa recommendation, Level B evidence) [5,13,26]. In prior guidelines, an aneurysmal growth rate ≥ 0.5 cm per year was also an indication for surgical treatment, though this is not explicitly recommended by current guidelines [25]. In patients meeting these criteria and with low operative risk, open arch replacement is recommended. Prior guidelines had also recommended that total arch replacement (TAR) is reasonable if patients have acute or chronic dissections that cause enlargement of the aortic arch (2010 AHA guidelines: Level B evidence) [25]. Figure 1 depicts one approach to a total arch replacement [27].

Guidelines also suggest that an arch repair can be considered in patients meeting the criteria for aneurysm repair in the adjacent ascending or descending aorta (EACTS/STS: Class I recommendation, Level C evidence) [13,26]. A hemiarch replacement, for example, is considered reasonable for patients undergoing surgery for an ascending aneurysm that extends into the proximal aortic arch [5,13]. If an aneurysm of the aortic arch extends into the proximal descending aorta, an elephant trunk (ET) procedure should be considered (EACTS/STS guidelines specifically suggest the frozen elephant procedure: Class IIa, Level B evidence); these include the conventional elephant trunk procedure and the frozen elephant trunk procedure, both of which are discussed in greater detail in a following section [5,13,26]. Moreover, guidelines specify that a distal anastomosis in arch zone 2 should be considered when performing either of the elephant trunk techniques (EACTS/STS: Class IIa recommendation, Level C evidence) [26]. If a patient with aortic arch aneurysm meets the criteria for surgical intervention but is at high risk for open repair, a hybrid or endovascular approach can be considered (EACTS/STS: Class IIb recommendation, Level C evidence) [5,13,26]. Features that make a patient at high risk for open repair include COPD, renal failure, previous stroke, coronary artery disease, and/or previous cardiac surgery [28]. Patients older than 75 years have also seen elevated complication and mortality rates [29]. At a broad level, when compared to the previous aortic guidelines from 2014, the new guidelines emphasize open surgical replacement in symptomatic patients at low or intermediate operative risk; they also recommend an elephant trunk procedure if an arch aneurysm extends into the descending aorta [13]. Table 4 summaries the surgical management recommendations based on isolated vs. multi-segment disease.

## 4. Operative Management Techniques

Treatment of aortic arch aneurysms generally fall into the open surgical, endovascular, or hybrid procedure categories. While open repair with TAR remains the gold standard for now, innovations in endovascular and hybrid techniques are progressively improving outcomes and addressing the drawbacks of open repair. Table 5 provides descriptions of the various approaches [4,5,30].

### 4.1. Open Repair vs. Hybrid and Endovascular Repair

Open repair of aortic arch aneurysms is of particular benefit in patients with large or symptomatic aneurysms, with acute aortic dissections in which the arch is involved, or with connective tissue disorders like Marfan syndrome [2,5,31]. However, open repair is relatively contraindicated in patients with advanced age or severe comorbidities who may be at higher surgical risk [28]. In such patients, hybrid or endovascular approaches are more appropriate.

These hybrid and endovascular approaches have been developed with the aim of reducing the perioperative morbidity and mortality seen in open TAR [32]. Current endovascular interventions fall into the following categories: (1) branched endovascular approach, where the graft is customized to have branches that connect to the supra-aortic arteries; (2) fenestrated endovascular approach, where the graft has fenestrations that line up with the supra-aortic arteries; and (3) parallel graft (PG) technique, where a side stent into the branching arteries (often called the “chimney” or “snorkel”) is placed parallel to the main graft in the aorta [33]. Though branched and fenestrated grafts are often preferred, they are custom-made devices and can have wait times up to three months; hence, parallel grafts or surgeon-modified fenestrated stent-grafts (SMFSGs) can be used in emergent situations [33].

Hybrid techniques, in turn, combine open and endovascular approaches [4]. These techniques typically involve open surgical debranching of the supra-aortic vessels, followed by TEVAR to exclude the aneurysm from the circulation [34]. In addition to high-risk anatomy or comorbidities that preclude open TAR, hybrid techniques may also be employed in patients where the aneurysm extends into the descending aorta [1]. Prior comparisons in outcomes between open surgical versus hybrid and endovascular approaches had shown no significant differences [5,35]. Yoshitake et al., for example, found that endovascular approaches saw shorter ICU and hospital stays than open surgical repair with no statistically significant difference in 30-day mortality (open vs. endovascular: 2.8% vs. 1.7%, respectively), 3-year survival (84.0% vs. 79.1%, respectively) and 5-year survival (78.3% vs. 72.4%, respectively) [36]. While reintervention was higher in the endovascular group (2.2% vs. 8.1%, respectively, at 5 years; *p*  =  0.04), rates of complications, including stroke, spinal cord deficits, renal failure, and pulmonary complications requiring tracheostomy, were not statistically different between open repair and endovascular approaches [36].

Recent studies, however, have shown more significant differences in outcomes. A large meta-analysis by Chen and group comparing outcomes between open and hybrid repair found that hybrid repair was associated with fewer perioperative complications, including reoperation for bleeding (open vs. hybrid repair: 8.07% vs. 3.96%, respectively; *p* = 0.01), postoperative pulmonary complications (14.75% vs. 5.02%, respectively; *p* < 0.0001), and acute renal failure (7.54% vs. 5.17%, respectively; *p* = 0.03) [37]. Open repair, however, saw lower rates of stroke (5.1% vs. 17.35%, respectively; *p* = 0.01), spinal cord ischemia (5.75% vs. 11.49%, respectively; *p* = 0.02), and permanent paraplegia (2.79% vs. 6.08%, respectively; *p* = 0.006). Thirty-day mortality and 1-year survival rates were not significantly different, but open repair showed better 3-year survival rates (hybrid vs. open repair hazard ratio: 1.69; *p* = 0.01) and 5-year survival rates (HR: 1.68; *p* = 0.01) [37]. These results were corroborated by Joo et al., who additionally found that open repair had higher 10-year survival rates (74.7% versus 42.6, respectively) [38]. A comparison of branched endovascular repair and open repair by Kawatou et al. found reintervention rates to be higher in the endovascular group than in the open repair group (open vs. endovascular repair: 3.3% vs. 26.8%, respectively; *p* < 0.05) [39]. Hence, the recent literature seems to suggest that hybrid and endovascular repairs decrease perioperative morbidity with similar short-term outcomes to open repair, but that open repair remains the most durable over the long-term.

Table 6 provides a summary of various studies evaluating outcomes of open surgical, hybrid, and endovascular repair [36,37,38,39,40,41,42,43,44,45,46,47,48,49,50,51,52,53,54,55,56]. The quality of the compiled evidence was variable, reflecting the heterogeneous study designs represented in this review. Ten cohort studies were amenable to Newcastle-Ottawa Scale (NOS) scoring, which is designed exclusively for primary observational cohort studies. NOS scores ranged from 7–9 out of 9, with six studies achieving a score of 9. Importantly, a perfect NOS score reflects methodological reporting standards rather than absence of selection bias; all included studies were retrospective, with treatment allocation driven by clinical risk profile. No randomized controlled trials exist in this area, and the overall body of evidence should be interpreted accordingly.

### 4.2. The Elephant Procedure

In certain situations, such as in acute dissections or when an arch aneurysm extends into the descending aorta, guidelines state that an elephant procedure may be considered [5]. There are two such procedures typically practiced—the conventional elephant trunk procedure (ET) and the frozen elephant trunk procedure (FET). The ET technique is a two-stage approach, whereas the FET technique is a single stage approach. In recent years, open total arch replacement has increasingly used the adjunct of either the ET or FET techniques [23]. A primary factor for deciding between ET and FET is whether the frozen elephant trunk can achieve an adequate distal seal. A second surgery may be required if the distal seal is not achieved, in which case the conventional elephant trunk may be preferred altogether [5].

Frozen elephant trunk devices, such as the Thoraflex™ Hybrid Graft (Terumo Aortic, Inchinnan, UK), typically integrate a woven surgical graft with a self-expanding stent-graft. This combination allows for a single-stage repair of the aortic arch and proximal descending aneurysms. Tan et al., in a review of 931 cases of frozen elephant trunk procedures, demonstrated an acceptable 30-day mortality of 0.6%, while overall mortality was 1.5%, and sustainable mid-term results [57]. Neurological injury occurred in 1.9% of cases, with stroke rates ranging from 5% to 10% and transient spinal ischemia was reported in approximately 2% to 5%. Most studies indicated no instances of permanent paraplegia. Reinterventions due to bleeding were necessary in 10% to 13% of cases, and renal injury was observed in 0.7% to 3% of cases, occasionally requiring temporary dialysis. The most encouraging finding was that nearly 100% of patients achieved positive aortic remodeling within 24 months, indicating successful thrombosis of the false lumen and expansion of the true lumen. Importantly, no confirmed cases of in-stent thrombosis or prosthesis-related leaks were documented, highlighting the device’s strong safety and durability profile in complex aortic arch repair [58,59]. FET offers significant advantages in specific situations. It promotes true lumen expansion and restores blood flow to the distal areas, serving as a lifesaving option for ruptures in the distal arch or proximal descending aorta.

Additionally, it reduces the risk of bleeding from the fragile false lumen. FET also minimizes the need for future interventions by providing a stable distal landing zone for secondary procedures. Furthermore, branched hybrid grafts enhance hemostasis through selective supra-aortic vessel reconstruction, improving overall procedural safety and durability [60].

Studies comparing ET and FET outcomes have traditionally shown that FET has significantly lower mortality rates but higher spinal cord ischemia rates. Hanif et al., for example, found that FET significantly reduced mortality (odds ratio: 0.55; 95% CI: 0.39–0.78) relative to conventional arch repair techniques, albeit with a significant increase in spinal cord ischemia (odds ratio: 2.20; 95% CI: 1.10–4.37) [61]. Leontyev et al. corroborated these results [62]. In addition to these results, Moula et al. found that mortality was 14.8% after ET and 7.9% after FET (*p* = 0.027) [63]. Spinal cord ischemia was found to be 2.7% after ET and 4.8% after FET (*p* = 0.004). No differences, however, were found in the incidence of stroke (ET vs. FET: 9.4% vs. 7.8%, respectively; *p* = 0.64) and renal failure (12.9% vs. 10.0%, respectively; *p* = 0.89) [63].

However, recent studies have called into question the higher spinal cord ischemic rates associated with FET. One analysis by Hage et al. found that the risk difference for spinal cord injury between ET and FET was not statistically significant (3.3%, 95% CI −0.4% to 7.0%, *p* = 0.085) [64]. Vernice et al. additionally found that FET saw higher 1-year survival than ET (hazard ratio: 0.63, 95% CI: [0.42; 0.95], *p*  =  0.03) with no significant increases in reintervention, stroke, spinal cord, ischemia, or renal failure [65]. Though more investigation is required, the current literature indicates that FET may have higher short-term and mid-term survival than ET with no significant increase in complication rates. It is for these reasons that the FET approach has progressively enjoyed greater use while usage of the ET approach has steadily decreased.

## 5. Hybrid Management Techniques

Hybrid repair with supra-aortic debranching and TEVAR remains a viable option for patients with aortic arch disease who are not candidates for complete endovascular repair. A review of 26 studies involving 956 patients reported a 30-day or in-hospital mortality rate of 11.9%, a stroke rate of 7.6%, and a 3.6% incidence of permanent spinal cord ischemia [66]. Eleshra et al. reported a 5% in-hospital mortality rate in their retrospective review of 112 patients who underwent hybrid arch repair in zones 0 to 2 [58]. They also found that early stroke incidence was higher in aneurysmal cases compared to dissecting cases. Previous reports indicated that patients in Zone 0 experienced poorer early and late outcomes than those in other zones [58]. Hiraoka et al. similarly observed no significant difference in 30-day mortality (4.7% vs. 7.0%, *p* = 0.41) but noted a higher incidence of lasting neurologic disability in the hybrid group (0% vs. 11.6%) [59].

**Figure 2:**
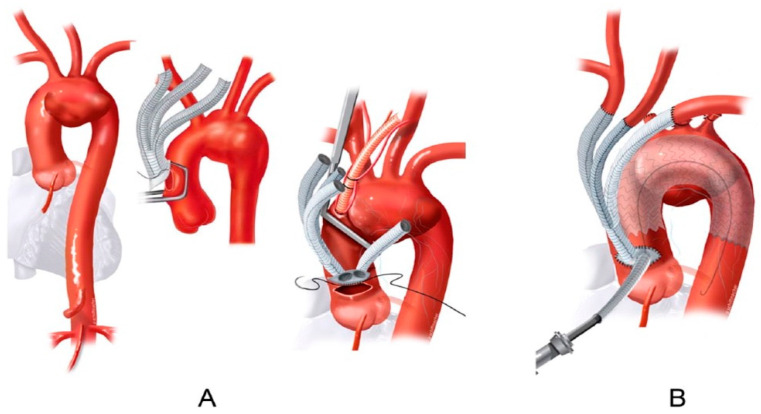
Type 1 hybrid aortic arch aneurysm repair. (**A**) Aneurysm pre-repair with operative anastomosis of the arch vessels to the proximal ascending aorta. (**B**) Deployment of a stent graft into the arch to exclude the aneurysm. Reprinted with permission from Sultan et al. [67], 2016 Sage Publishing.

The higher rates of neurologic complications in hybrid repairs are due to the combination of a transient loss of cerebral perfusion and extensive manipulation of the supra-aortic branches leading to increased risk of embolism [60]. In particular, the likelihood of stroke during TEVAR is primarily determined by the proximity of the landing zone, with Zone 0 presenting the highest risk because of the increased manipulation around the supra-aortic vessels [67]. Risk mitigation strategies emphasize meticulous preoperative evaluation, intraoperative cerebral protection, and individualized patient selection [67,68]. These include avoiding Zone 0 TEVAR in high-risk patients unless necessary, and applying stricter criteria for elective interventions in this zone. Comprehensive preoperative screening for arch atheroma and cerebrovascular disease is essential. During the procedure, minimizing arch manipulation and reducing operative time are key principles. Choosing the appropriate revascularization technique based on both the landing zone and patient-specific anatomy further improves safety [67]. The key to improving outcomes lies in careful patient selection and the continued advancement of endovascular technology. Figure 2 depicts an aortic arch aneurysm before and after hybrid repair. Table 7 summarizes the aforementioned neurologic risk considerations according to the aortic arch landing zone.

## 6. Endovascular Techniques

In the last two decades, endovascular repair for aortic arch aneurysms has emerged as the first-line treatment for diseases of the descending aorta, with its indications expanding due to the significant morbidity and mortality of open surgery. The current approaches, including multi-branched and inner-branched endografts, single-branch modular systems, custom fenestrated or physician-modified grafts, and additional techniques like chimney, periscope, and in situ fenestration, have shown high success rates, instilling confidence in their efficacy [69].

### 6.1. Branched, Fenestrated, and Parallel Endovascular Approaches

Multiple studies have compared outcomes between different endovascular techniques, with no conclusive differences being found. Spath et al. conducted a meta-analysis comparing branched and fenestrated endografts; they found similar rates of technical success, 30-day mortality, strokes, permanent, paraplegia, spinal cord, ischemia, renal failure, and cardiac events [30]. Fenestrated endovascular repair, however, saw higher rates of endoleak (fenestrated vs. branched: 9.8% vs. 2.6%, respectively; *p* = 0.034). 1-year survival rates ranged from 82 to 96.4%, 3-year survival rates ranged from 75 to 90.8%, and 5-year survival rates ranged from 80.8 to 84.4% [30]. These mid-term survival rates are all higher than those found in other studies; Nana et al., for example, reported 3-year survival rates of 67% and 5-year survival of 59% [70]. Though they reported an incidence of adverse aortic events—open surgical repair, graft infection, branch vessel occlusion, aneurysm enlargement—of 24% at five years, what is promising is that freedom from aortic related death at five years was 93% [70]. Thus, current data indicates that fenestrated and branched endovascular repair offer reduced early mortality with varying outcomes in the longer term.

Studies comparing parallel and branched endovascular repair have found similar outcomes. One study by Kudo et al. found similar rates of 30 day mortality, stroke, and spinal cord ischemia between parallel and branched endovascular repair [71]. Endoleak rates were significantly higher in patients who got parallel TEVAR (parallel vs. branched: 40% vs. 0%, respectively; *p* = 0.001). Moreover, the 8-year survival rates for the two groups were 32.3% and 70.9%, respectively, though the difference did not reach statistical significance (*p* = 0.054) [71]. In short, the current literature indicates that branched, fenestrated, and parallel TEVAR have similar outcomes with increased endoleak rates in the fenestrated and parallel approaches, though more investigation is required.

Of note, use of intraoperative TEE has been reported to monitor guidewire and device positioning, detection of atheroma or thrombus, and early identification of complications such as endoleak and pericardial effusion [72].

### 6.2. Off-the-Shelf Modular and Single-Branch Systems: NEXUS Duo and NEXUS TRE

**Figure 3:**
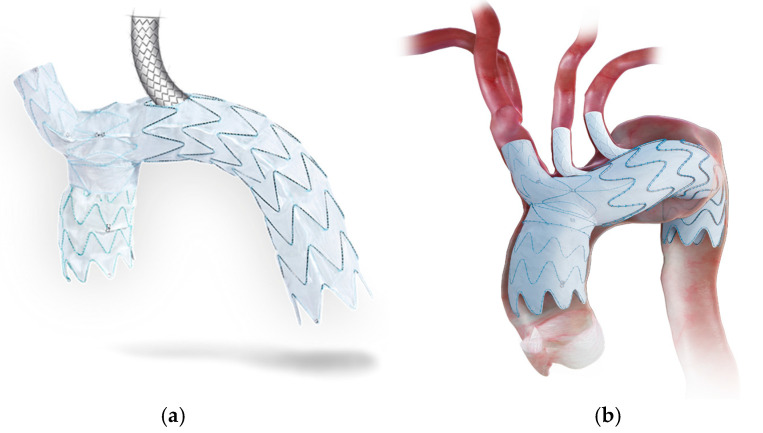
(**a**,**b**) NEUXUS Duo and TRE aortic arch endovascular device system in use for the Management of aortic arch aneurysm.

The NEXUS Duo Aortic Arch Stent Graft System (Endospan, Herzliya, Israel) with a second branch channel allows transfemoral introduction of all stent grafts necessary for complete endovascular arch repair, with minimal manipulation of supra-aortic vessels (Figure 3a). It is specifically intended for high-risk patients who require zone-0 repair. The device’s unique features include a proximal ascending module, a distal arch component, and a self-aligning junction that connects the two. The current single-branch Nexus is off-the-shelf and available for patients requiring urgent life-saving treatment. A side branch ensures continuous blood flow to the supra-aortic vessels. In a prospective multicenter study by D’Onofrio et al., the device demonstrated a technical success rate of 100%. Survival rates at one and three years were 89% and 71%, and unplanned reinterventions occurred in 11% and 29% of cases [73]. NEXUS TRE™ Aortic Arch Stent Graft is a custom-made device which eliminates the need for supra-aortic debranching (Figure 3b). However, presently it is approved for use outside US only.

### 6.3. GORE^®^ TAG^®^ Thoracic Branch Endoprosthesis (TBE)

The GORE^®^ TAG^®^ Thoracic Branch Endoprosthesis (TBE) is a single-branch, off-the-shelf endograft with promise for improving patient outcomes. Engineered to preserve left subclavian artery (LSA) perfusion and facilitate complete endovascular repair of zone 0 to 2 thoracic aortic pathologies without the need for additional open revascularization, the TBE has shown significant potential. Recent institutional data (2022–2023) from 38 patients demonstrated 100% technical success and no major device-related complications, with all side branches patent and no reinterventions at early follow-up [74]. In a recent multicenter feasibility trial, which enrolled 40 patients (31 zone 2; 9 zone 0/1), over three years of follow-up, neither group had device migration, fracture, or aortic rupture cases. In the zone 2 group, 97% of patients did not need reintervention at one and three years, though two side branch occlusions occurred. Two patients had aneurysm growth greater than 5 mm, but there was no endoleak or need for reintervention. Survival rates were 90% at one year and 84% at three years. In the zone 0/1 group, there were no reinterventions, loss of branch patency, or aneurysm growth at three years. Three patients had cerebrovascular events during follow-up: two were unrelated to the device or treatment, and one could not be clearly linked. Two patients in this group died from causes unrelated to the procedure or aneurysm. These consistent outcomes highlight TBE as a durable and safe option with good mid-term patency for high-risk zone 0–2 aortic repair. However, further larger cohorts are required to confirm the safety and efficacy of the device [75]. Of interest, Rizza et al. described their experience with the Castor single-branched thoracic aortic stent grafts, which are used to maintain perfusion of the left subclavian artery in zone 2 arch repairs. They found no complications or endoleak in the 10 patients, and perfusion of the left subclavian artery remained sufficient in all patients [76]. A follow-up study used the same stent-graft across multiple Italian centers to achieve proximal sealing in zone 1 or 2. The investigators saw 3.9% rate of minor embolic stroke with no operative deaths or major adverse events. 5.8% of cases had intraoperative type 1a endoleak, and graft patency rate was 100% at a median follow-up of 22 months [77]. This graft is not approved for use in the USA, though trials are currently underway.

### 6.4. Custom and Physician-Modified Endografts

The development of custom and physician-modified endografts (PMEGs) has broadened the range of endovascular treatments available for complex aortic arch aneurysms, particularly in patients who are not suitable candidates for open surgical repair. Custom-manufactured fenestrated and branched grafts (such as RelayBranch and E-nside) provide precise anatomical matching and long-lasting outcomes; however, they have significant production durations, rendering them unsuitable for urgent circumstances. PMEGs, generated intraoperatively from conventional stent grafts, allow for fast therapy and have shown great technical success (>90%) and acceptable perioperative mortality (5–10%), with stroke being the predominant consequence [30,78]. Current guidelines recommend using these techniques in specialized, high-volume centers that employ careful preoperative imaging, cerebral protection, and multidisciplinary planning. Collectively, these advances constitute a substantial progression toward the realization of safe, fully endovascular solutions for the repair of complex aortic arch aneurysms [5].

### 6.5. Adjunctive Strategies: Chimney/Periscope Techniques and In Situ Fenestration

The chimney technique was initially used to save the aortic arch’s accidentally covered left subclavian artery [68]. This method is predicated on implanting multiple stent-grafts and parallel covered stents [79]. In a previous meta-analysis of 379 patients, the technical success rate was 91%. Thirty-day mortality was 4%, patency was 93%, perioperative endoleak was 21% and stroke was 5% after patients received the chimney technique treatment [80]. According to the current study, the chimney technique is safe and effective for treating aortic arch diseases in various aortic zones in both elective and emergency cases. Larger series results and comparative studies are required to ascertain safety and efficacy, as the long-term effects are unknown [79,80].

In situ fenestrations have also been implemented in appropriate situations, particularly in emergent situations in which patients are not suited for open repair. One systematic review by Houérou et al. found that in situ fenestrated endovascular repair saw a technical success rate of 98% [56]. Stroke occurred in 4.5% and mortality was observed in 3.2% of patients (mean follow-up = 15 months). These results were corroborated by a recent study by Li et al., which found a technical success rate of 100% and overall mortality of 8.7%; reintervention was required in 7.8% of patients [81].

Overall, endovascular device selection in clinical practice is largely driven by urgency and anatomic complexity rather than theoretical device superiority. In unstable or urgent cases, off-the-shelf systems such as the NEXUS™ (Endospan), NEXUS Duo (Endospan) and GORE^®^ TAG^®^ Thoracic Branch Endoprosthesis (TBE), as well as physician-modified endografts (PMEGs) and adjunctive techniques such as chimney, periscope, or in situ fenestration, are favored due to immediate availability, accepting higher risks of neurologic events and endoleak. In stable, elective settings, custom-manufactured branched or fenestrated endografts and hybrid open–endovascular solutions such as the Thoraflex™ Hybrid (Terumo Aortic) are preferred for superior anatomic conformity and long-term durability. Hybrid debranching plus TEVAR remains an important strategy for high-risk patients who need intervention urgently. Table 8 summarizes the endoleak incidence by endovascular approach and devices.

### 6.6. Overall Outcomes, Future Directions, and Decision-Making

Based on the above discussion, it is apparent that endovascular repair of the aortic arch has consistently demonstrated high technical success rates, exceeding 90%, and acceptable mid-term survival rates. This method clearly shows advantages in early outcomes compared to traditional open repair. The safety and effectiveness of devices such as NEXUS™ and GORE^®^ TAG^®^ TBE are well-established, providing durable aneurysm exclusion and low reintervention rates. Custom and physician-modified grafts effectively address complex anatomical challenges, although they carry a persistent risk of stroke. While hybrid arch repair is effective, it is associated with higher rates of neurologic complications and mortality, especially in interventions targeting zone 0. Adjunctive techniques, such as chimney and in situ fenestration, expand the applicability of these repairs but require further validation for long-term durability. Modern endovascular strategies have greatly improved the safety and feasibility of managing complex aortic arch pathologies, contingent upon careful patient selection and specialized procedural expertise.

The existing evidence largely stems from prospective single-arm IDE trials, multicenter registries, and observational studies. There is a notable absence of direct randomized comparisons with traditional open aortic arch replacement procedures. Key areas for future focus include: (1) the development of larger multicenter cohorts with consistent outcome metrics, (2) feasibility studies comparing different device types directly, (3) initiatives and clinical trials aimed at minimizing the incidence of stroke, and (4) extended follow-up periods to evaluate the durability of devices and the necessity for reintervention beyond a timeframe of 3 to 5 years. Ongoing advancements in device design, cerebral protection methods, and procedural techniques are expected to influence the integration of endovascular arch repair over the next decade.

With regard to decision-making, simply comparing the open surgical, hybrid, or endovascular management options is not the best approach. Rather, determining the best option for each individual patient requires the consideration of the patient’s aortic anatomy and broader medical needs. Open surgery will likely be the best option for younger, healthier patients with fewer comorbidities, or those with genetically-based aortic diseases. Hybrid management is better suited for patients with a proximal landing zone that can be used for supra-aortic debranching in combination with TEVAR. Lastly, endovascular aortic arch repair is more favorable in older patients with relevant comorbidities. While it currently requires a proximal safe fixation zone, it represents a very promising option for the future.

## 7. Limitations and Conclusions

### 7.1. Limitations

There are several limitations to this study. First, as a narrative review, it is subject to inherent selection bias, as the literature selection did not follow a strict systematic search protocol. Second, the available evidence on aortic arch aneurysm management heavily relies on retrospective surgical series, which are susceptible to publication bias and may overrepresent outcomes from high-volume, specialized centers. Furthermore, the cited studies frequently feature short and highly variable follow-up periods, limiting our ability to draw definitive conclusions regarding long-term graft durability, late endoleaks, or delayed neurological events. Finally, there is a notable absence of randomized controlled trial (RCT) data comparing open, hybrid, and endovascular repair strategies. Consequently, current management recommendations remain guided primarily by observational data and expert consensus, highlighting the critical need for prospective, multicenter registries to establish more robust clinical guidelines.

### 7.2. Conclusions

Management of isolated aortic arch aneurysms remains a complex challenge, requiring a strategic approach to prevent life-threatening complications such as dissection or rupture. The evolution of surgical, hybrid, and endovascular techniques has shifted the treatment paradigm away from a “one-size-fits-all” model toward a more personalized strategy; the selection of the optimal approach should be dictated by the patient’s pathogenetic background (such as connective tissue disorders), the anatomic extent of the disease, patient age, and the presence of related comorbidities. While open repair remains the gold standard for younger, healthier patients, hybrid and endovascular technologies have provided viable management modalities for older patients or those with significant open surgical risk. Ultimately, the continued development of technology and technique offers the promise of improved endovascular success in the treatment of these aneurysms.

## Figures and Tables

**Table 1:** Genetic and acquired etiologies of aortic arch aneurysms, classified with mechanisms and supporting references.

**Genetic Causes**		
Etiology	Mechanism	% of Aortic Arch Aneurysms
Marfan syndrome	FBN1 mutation → defective fibrillin-1 → medial degeneration of aortic wall	<5%
Loeys-Dietz syndrome	TGFBR1/2, SMAD3, TGFB2 mutations → abnormal TGF-β signaling → aggressive aneurysm progression	<5%
Ehlers–Danlos syndrome	COL3A1 mutation → fragile connective tissue, aneurysm/dissection risk	<1%
Familial thoracic aortic aneurysm/dissection (FTAAD)	Non-syndromic, linked to ACTA2, MYH11, MYLK, PRKG1 mutations	~5%
Bicuspid aortic valve (BAV) aortopathy	Genetic predisposition and abnormal flow dynamics → aortic arch dilation	~5–10%
**Acquired Causes**		
Etiology	Mechanism	% of Aortic Arch Aneurysms
Atherosclerosis	Degenerative changes in intima/media from lipid deposition and inflammation	60–70%
Inflammatory aortitis	Giant cell arteritis, Takayasu arteritis, idiopathic aortitis cause wall weakening	~2–5%
Infectious (mycotic) aneurysms	Due to bacteria (Staphylococcus, Salmonella), syphilitic aortitis	<2%
Trauma	Blunt thoracic trauma, iatrogenic post-surgical/endovascular injury	<1%

**Table 2:** Imaging techniques for the evaluation and management of aortic arch aneurysms. Sensitivity and specificity values for CT, MRI/MRA, and chest X-ray represent data for thoracic aortic disease, encompassing aortic arch aneurysms and dissections.

Imaging Technique	Utility	Advantages	Disadvantages and Risks	Sensitivity and Specificity
Transthoracic echocardiography (TTE)	1st line; initial evaluation at bedside.	Rapid and widely available with no radiation exposure.	Operator-dependent and hence may have limited view of the arch and aorta.	Sensitivity: 79% Specificity: 88%
CT angiography (CTA), often ECG-gated	2nd line; definitive assessment and pre-op planning.	Excellent spatial resolution with 3D reconstruction.	Exposure to radiation and iodinated contrast.	Sensitivity: 100% Specificity: 100%
Magnetic resonance imaging (MRI/MRA)	3rd line or alternative to CTA.	Accurate characterization of diameters and flow; no radiation exposure, thus good for serial surveillance.	Longer scan than CT, concerns about gadolinium exposure in renal failure.	Sensitivity: 98% Specificity: 98%
Transesophageal echocardiography (TEE)	2nd/3rd line; often used for intraoperative/procedural guidance.	High sensitivity for ascending aorta.	Semi-invasive requiring sedation.	Sensitivity: 91% Specificity: 99%
Chest radiograph (CXR)	Adjunctive, triage	Fast and inexpensive	Cannot be used to rule out aneurysm/dissection or to plan therapy.	Sensitivity: 71% Specificity: Variable

**Table 3:** Summary of medical management in aortic arch aneurysms.

Strategy	Recommendation	Rationale and Notes
Blood pressure control	Target < 130/80–140/90 mmHg using β-blockers, ACE inhibitors, or ARBs	Decreases aneurysm growth, β-blockers preferred in Marfan syndrome
Lipid management	LDL-C < 55 mg/dL with statin therapy	Reduces atherosclerotic risk
Smoking cessation	Complete abstinence advised	Decreases aneurysm growth rate
Imaging surveillance (<4.0 cm)	CTA or MRA every 12 months (if aneurysm < 4.0 cm) or every 6 months (if >4.0 cm)	Evaluate aneurysm growth

**Table 4:** Summary of surgical management in aortic arch aneurysms, grouped by involvement of different segments of the aorta.

Category	Indications and Recommendations	Preferred Approach
Isolated arch disease	Symptomatic, aneurysm ≥ 5.5 cm, and acute or chronic dissections	Open total arch replacement
Multi-segment disease involving the ascending or descending aorta	Ascending aneurysm that extends into the proximal aortic arch, or an aneurysm of the aortic arch extends into the proximal descending aorta	Frozen elephant trunk with distal anastomosis in arch zone 2 if descending aorta is involved Hemiarch replacement if only ascending aorta involved
High-risk patients	Severe comorbidities, including COPD, renal failure, previous stroke, coronary artery disease, and/or previous cardiac surgery	Hybrid or branched endovascular repair

**Table 5:** Contemporary treatment options for aortic arch aneurysms grouped by open, endovascular, and hybrid approaches.

Category	Technique	Description
Open repair	Total arch replacement (TAR)	Surgical replacement of the aortic arch with a graft and subsequent reimplantation of brachiocephalic vessels Gold standard for aortic arch aneurysm repair
	Zone-2 arch replacement	Less extensive open replacement ending at zone 2 May include head-vessel reimplantation and debranching Creates a durable proximal surgical landing zone for later TEVAR May leave residual disease distal to graft
	Conventional elephant trunk (ET)	Two-staged approach for aneurysms involving majority of the thoracic aorta Proximal aorta and arch are surgically replaced, with the distal end of the graft (the “elephant trunk”) hanging freely in the descending aorta for later repair Involves interval risk of rupture/complication before completion in second stage
Hybrid repair	Surgical debranching + TEVAR	Surgical debranching of arch vessels followed by placement of stent graft Appropriate for high-risk patients who cannot tolerate full TAR
	Frozen elephant trunk (FET)	Open arch replacement that combines a standard total open arch repair with distal endovascular repair that may be conducted simultaneously or later Appropriate for complex dissections/aneurysms Spinal cord ischemia risk, stroke risk, still major open operation.
Endovascular repair	Branched or fenestrated grafts	Endografts with branches or fenestrations implanted to preserve flow to head vessels New devices and grafts available with promising early/mid-term longevity

**Table 6:** Published studies and their results comparing outcomes between open surgical and endovascular or hybrid repair of aortic arch aneurysms.

Lead Author	Publication Year (Study Period)	Center	Indication	Approach	Result	Newcastle-Ottawa Scale Score
Chen [37]	2024	N/A, meta-analysis of sixteen studies		Open vs. hybrid	Hybrid had fewer perioperative bleeding (open vs. hybrid repair: 8.07% vs. 3.96%, respectively; *p* = 0.01), postoperative pulmonary (14.75% vs. 5.02%, respectively; *p* < 0.0001), and renal complications (7.54% vs. 5.17%, respectively; *p* = 0.03); open repair saw fewer neurologic complications (5.1% vs. 17.35%, respectively; *p* = 0.01) and better 3- (hybrid vs. open repair hazard ratio: 1.69; *p* = 0.01) and 5-year survival rates (HR: 1.68; *p* = 0.01).	
Wen [51]	2024	Multicenter Study	Aortic Arch Aneurysm	Fenestrated endovascular repair	Fenestrated endovascular repair in zone 0 saw 94.2% survival and 81.8% freedom from secondary intervention at one year.	
Houérou [56]	2023	N/A, systematic analysis of 6 studies	Aortic Arch Aneurysm	In situ fenestrated endovascular grafts	In situ fenestrated endovascular repair saw a technical success rate of 98%. Stroke occurred in 4.5%. Mortality was observed in 3.2% of patients (mean follow-up = 15 months).	
Planer [54]	2023	Multicenter Study	Aortic Arch Aneurysm	NEXUS endovascular graft	The NEXUS Aortic Arch Stent Graft System saw a 100% procedural success rate, with one-year survival of 89.3% and a stroke rate of 3.6%.	
Zhan [47]	2021	N/A, meta-analysis of five studies		Open vs. hybrid	Open repair had significantly superior 1-year (OR 0.42; 95% CI: 0.20–0.88; *p* = 0.02) and 2-year (OR 0.48; 95% CI: 0.26–0.88; *p* = 0.02) survival rates, and hybrid repair saw significantly higher stroke rates (*p* = 0.0004). Other outcomes had no significant differences.	
Tenorio [53]	2021 (2016–2019)	Multicenter Study	Aortic Arch Aneurysm	Branched endovascular graft	3-vessel inner-branch endovascular grafts saw survival and stroke rates of 90% and 5%, respectively; this was similar to rates that have been seen in open surgical repair in high-risk patients, though the rate of secondary interventions was high (31%).	
Spanos [55]	2021	Multicenter Study		Branched endovascular graft	The 3-inner-branch Cook Zenith Arch endograft was found to have a 79% suitability rate, particularly in anatomy with a 90 mm distance between the opening of the first and third inner branches.	
Joo [38]	2019 (2002–2017)	Yonsei University College of Medicine	Aortic Aneurysm	Open vs. hybrid	Hybrid and open repair had similar in-hospital mortalities with open repair seeing fewer stroke rates and superior survival rates at 5 and 10 years (42.6% versus 74.7%, respectively; *p* = 0.043).	9/9
Gokalp [40]	2018 (2004–2010)	İzmir Katip Celebi University	Aortic Arch Aneurysm	Open vs. hybrid	There was no significant difference in neurologic events or survival at 1 and 5 years. Open repair required more postoperative revision (*p* = 0.01) and ventilation (*p* = 0.017).	7/9
Chakos [48]	2018	N/A, meta-analysis of nine studies		Open vs. hybrid	Hybrid repair saw significantly higher survival rates at 1, 2, 3, 5, and 7 years on pooled Kaplan–Meier analysis [HR 0.82 (0.69; 0.99), *p* = 0.04], though this was sensitive to the results of one specific study. Exclusion of the particular study yielded results that favored survival in the open repair group.	
Miao [49]	2017	N/A, meta-analysis of seven studies		Open vs. hybrid	Hybrid and open repair did not have significant differences in operative mortality, late mortality, neurologic deficits, though hybrid repair did see higher reintervention rates (OR 3.43; 95% CI 1.72–6.84; *p* = 0.0005).	
Hori [41]	2017 (2008–2016)	Saitama Medical Center	Isolated Aortic Arch Aneurysm	Open vs. endovascular	Open repair had superior mid-term survival rates (*p* < 0.001) and freedom from reintervention (*p* = 0.009).	9/9
Kawatou [39]	2017 (2007–2014)	Kyoto University Graduate School of Medicine	Aortic Arch Aneurysm	Open vs. branched endovascular	Endovascular approach had higher reintervention rates (open vs. endovascular repair: 3.3% vs. 26.8%, respectively; *p* < 0.05) with similar survival through 5 years.	7/9
Yoshitake [36]	2017 (2001–2016)	Keio University School of Medicine	Aortic Aneurysm	Open vs. endovascular	Endovascular repair saw shorter ICU and hospital stays (*p* < 0.001 for both) with no significant difference in 30-day mortality, 3-year survival, 5-year survival, and postoperative complications.	9/9
Spear [52]	2017	Hospital Center University De Lille		Branched endovascular grafts	Custom-made 3-inner-branched endovascular grafts yielded encouraging perioperative results and were patent at 6 months in patients at high risk for open surgery.	
Cazavet [43]	2016 (2002–2014)	University Hospital of Toulouse	Aortic Arch Aneurysm	Open vs. hybrid	Hybrid and open repair did not have significant differences in in-hospital mortality, nor in survival at 1, 3, 5, and 7 years. Open repair had significantly lower reintervention rates (*p* = 0.045, 95 CI: 0.06–0.97).	7/9
Tokuda [44]	2016 (2002–2014)	Nagoya University Graduate School of Medicine	Aortic Arch Aneurysm	Open vs. hybrid	Both groups had similar short-term mortality rates, but hybrid repair saw a lower rate of freedom from aortic events than the open repair group (79 and 99% at 24 months, respectively; *p* < 0.0001).	9/9
De Rango [42]	2015 (2007–2013)	Hospital S. Camillo-Forlanini, Rome	Aortic Arch Aneurysm	Open vs. endovascular	Endovascular and open repair had similar 30-day mortality and 4-year survival rates.	7/9
Iba [45]	2014 (2008–2013)	National Cerebral and Cardiovascular Center, Japan	Aortic Arch Aneurysm	Open vs. hybrid	There was no significant difference in early mortality and survival at 3 years between the 2 groups; the hybrid group saw shorter ICU stays (open vs. hybrid; 4.7 vs. 1.6 days, *p* = 0.018) but higher reintervention rates at 3 years (1% vs. 20%, respectively, *p* < 0.001).	9/9
Sood [46]	2014 (1993–2013)	University of Michigan	Isolated Aortic Arch Aneurysm	Open vs. endovascular	Endovascular and open repair had no significant differences in 5-year survival, but endovascular had higher reintervention rates (open 94% vs. endovascular or hybrid 78% at 2 years; *p* = 0.018).	9/9
Benedetto [50]	2013	N/A, meta-analysis of four studies		Open vs. hybrid	Hybrid and open repair had similar operative mortality rates and neurologic deficits postoperatively.	

**Table 7:** Zone-stratified neurologic risk considerations in aortic arch repair.

Landing Zone	Typical Techniques	Neurologic Risk Profile	Key Considerations
Zone 0	Total arch debranching + TEVAR, branched endografts, FET	Highest stroke risk; increased embolic burden and cerebral manipulation; higher SCI risk in extensive coverage	Requires manipulation near supra-aortic vessels and ascending aorta; careful cerebral protection and patient selection essential
Zone 1	Partial debranching + TEVAR	Intermediate stroke risk	Reduced arch manipulation compared with Zone 0 but still requires supra-aortic vessel intervention
Zone 2	Left subclavian artery coverage with/without revascularization; single-branch devices	Lowest neurologic complication rates among arch endovascular repairs	Often technically simpler with lower embolic burden and shorter procedural duration

**Table 8:** Reported endoleak incidence after endovascular and hybrid aortic arch repair.

Technique/Device	Reported Endoleak Rate	Follow-Up Duration	Comments
Branched endografts	~2.6%	Mid-term follow-up	Lower endoleak rates compared with fenestrated techniques
Fenestrated endografts	~9.8%	Mid-term follow-up	Increased risk due to fenestration alignment challenges
Parallel graft/chimney techniques	Up to 21–40%	Perioperative to long-term	Higher gutter-related endoleak incidence
In situ fenestration	Variable; low early incidence reported	Mean follow-up ~15 months	Limited long-term durability data
GORE^®^ TAG^®^ TBE	No endoleak reported in early/mid-term studies	Up to 3 years	Limited cohort sizes
NEXUS systems	Low reported endoleak incidence	1–3 years	Further long-term data needed
Hybrid debranching + TEVAR	Variable across studies	Heterogeneous	Reporting inconsistent between series

## Data Availability

No new data were created or analyzed in this study.

## Abbreviations

FET	Frozen elephant trunk
CTA	Computed tomography angiography
MRA	Magnetic resonance angiography
TBE	Thoracic branch endoprosthesis
TTE	Transthoracic echocardiography
TEE	Transesophageal echocardiography
TEVAR	Thoracic endovascular aortic repair
